# Breast mass as the first sign of metastasis from rectal carcinoma: a case report and review of the literature

**DOI:** 10.3389/fonc.2023.1211645

**Published:** 2023-06-26

**Authors:** Jiawei Xu, Chao Liu, Chengdong Yu, Tenghua Yu, Fan Fan, Xiaofang Zhang, Chuansheng Huang, Wen Chen, Zhengkui Sun, Meng Zhou

**Affiliations:** ^1^ Department of Breast Surgery, Jiangxi Cancer Hospital, The Second Affiliated Hospital of Nanchang Medical College, Jiangxi Clinical Research Center for Cancer, Affiliated Cancer Hospital of Nanchang University, Nanchang, Jiangxi, China; ^2^ Department of Pathology, Jiangxi Cancer Hospital, The Second Affiliated Hospital of Nanchang Medical College, Jiangxi Clinical Research Center for Cancer, Affiliated Cancer Hospital of Nanchang University, Nanchang, Jiangxi, China

**Keywords:** rectal neoplasms, breast tumor, neoplasm metastases, recurrent, case report

## Abstract

We present a case report of a 41-year-old woman who developed a left breast mass 18 months after undergoing Dixon rectal cancer surgery. The purpose of this case report is to highlight the possibility of breast metastases in patients with colorectal cancer and emphasize the importance of careful evaluation and follow-up as well as timely and accurate diagnosis and management of the metastatic disease. During the physical examination in 2021, we noted that the lower border of the mass was 9 cm from the anal verge and that it occupied approximately one-third of the intestinal lumen. A pathological biopsy revealed the mass in the patient’s intestinal lumen was a rectal adenocarcinoma. The patient underwent Dixon surgery for rectal cancer and received subsequent chemotherapy. The patient had no prior history of breast-related medical conditions or a family history of breast cancer. During the current physical examination, we discovered multiple lymphadenopathies in the patient’s left neck, bilateral axillae, and left inguinal region, but none elsewhere. We observed a large erythema of about 15x10 cm on the patient’s left breast, with scattered hard nodes of varying sizes. Palpation of the area beyond the upper left breast revealed a mass measuring 3x3 cm. We conducted further examinations of the patient, which revealed the breast mass and lymphadenopathy on imaging. However, we did not find any other imaging that had significant diagnostic value. Based on the patient’s conventional pathology and immunohistochemical findings, combined with the patient’s past medical history, we strongly suspected that the patient’s breast mass was of rectal origin. This was confirmed by the abdominal CT performed afterward. The patient was treated with a chemotherapy regimen consisting of irinotecan 260 mg, fluorouracil 2.25 g, and cetuximab 700 mg IV drip, which resulted in a favorable clinical response. This case illustrates that colorectal cancer can metastasize to unusual sites and underscores the importance of thorough evaluation and follow-up, particularly when symptoms are atypical. It also highlights the importance of timely and accurate diagnosis and management of metastatic disease to improve the patient’s prognosis.

## Case

**Figure 1:**
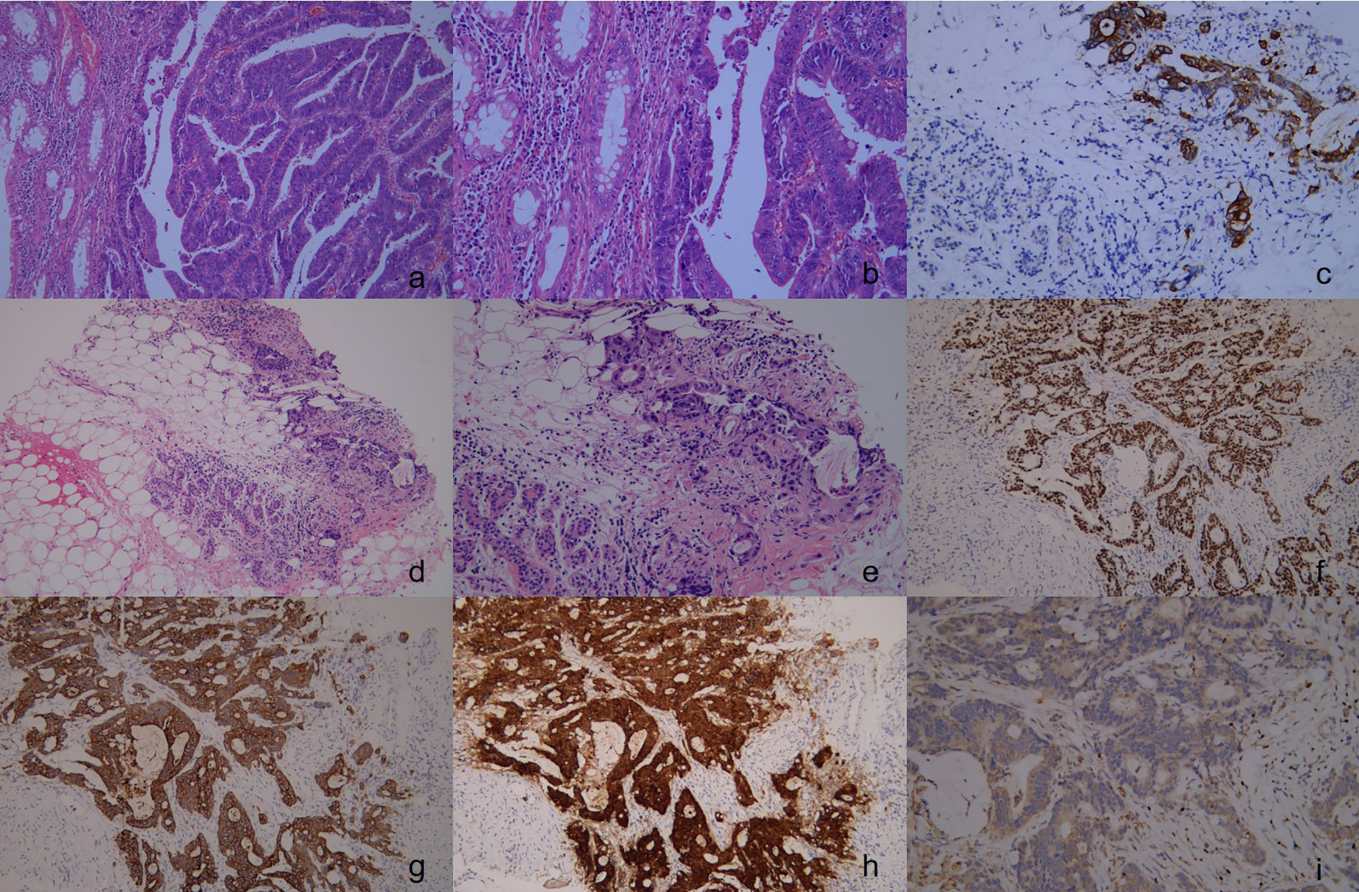
The pathology and immunohistochemical staining of rectal cancer specimens in the first operation. Show an irregular glandular growth pattern with intraglandular necrotic debris, with a large nucleus, hyperchromatic nuclei, obvious nuclear atypia, cytoplasmic depletion, red staining, and invasive growth. (**(A)**, H&E stain, ×100), (**(B)**, H&E stain, ×200), Immunohistochemical staining revealed that the rectal cancer cells were positive for CK20 (**(C)** ×100). Upon pathological examination of the breast tumor specimen, malignant cells were observed along with normal breast tissue. The glandular epithelium was found to proliferate into papillary and tubular structures, with large nuclei, with hyperchromatic nuclei, obvious nuclear -atypia, and cytoplasmic depletion. The cells exhibited invasive growth, with no carcinoma in situ component detected. (**D**, H&E stain, ×100), (**E**, H&E stain, ×200), Immunohistochemical staining revealed that the breast tumor cells were positive for CK20 (**F** ×100), CDX-2 (**G** ×100), Villin (**H** ×100), and were negative for GATA-3 (**I** ×100).

Without any previous history of breast-related illness or family history of breast cancer, a 41-year-old female patient presented at our hospital with a lump in her left breast that had been present for 2 months. In 2021, the patient underwent a physical examination, during which it was noted that the lower border of the mass was 9 cm from the anal verge and occupied approximately one-third of the intestinal lumen. The patient’s initial treatment for rectal cancer was completed at Wannian County People’s Hospital. A pathological biopsy confirmed the presence of rectal low-differentiated adenocarcinoma, which was graded as rectal adenocarcinoma stage III (T3N2M0) (**Figure 1**). The immunostaining results showed CDX-2(+), CK8/18(+), CgA(-), Syn(-), Her-2(1+), CD34(suggestive of vascular tumor embolus), D2-40(+), S-100(suggestive of nerve invasion), CK20(+), and Ki-67 about 90% (+). After undergoing Dixon surgery for rectal cancer, the patient received subsequent chemotherapy without prior neoadjuvant therapy. The exact regimen and doses of the patient’s chemotherapy consisted of 5 courses of bevacizumab 400 mg along with oxaliplatin 200 mg IV drip after surgery, and oral capecitab 1.5 g twice daily for 14 consecutive days. Every 21 days is a cycle. The patient with rectal cancer did not receive radiotherapy. The reason for not proceeding with radiotherapy was that the patient strongly rejected this treatment option. However, patient’s initial treatment for rectal cancer was considered successful until she presented with a lump in her left breast 18 months later.

**Figure 2:**
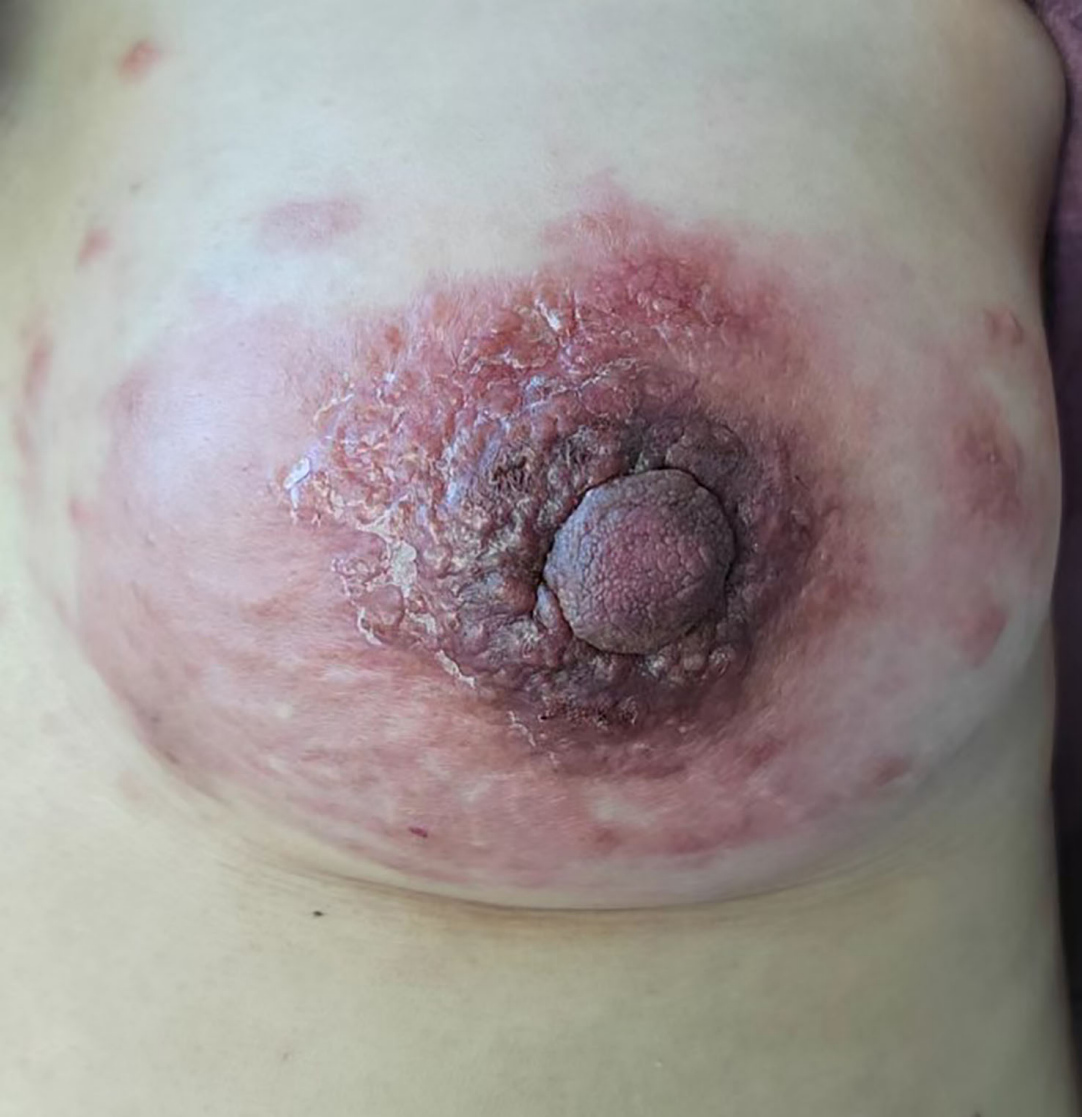
Large redness and swelling visible in the left breast at the time of presentation.

Physical examination revealed multiple enlarged lymph nodes in the left neck, bilateral armpits, and left groin area. A large red swollen area of approximately 15 x 10 cm was observed in the left breast with hard nodules of various sizes scattered around it (**Figure 2**). Palpation of the left breast also revealed a 3 x 3 cm lump outside the upper part. The patient’s other physical examinations were negative, and the tumor markers (CEA, CA 19-9, CA 15-3) were all in the normal range.

**Figure 3:**
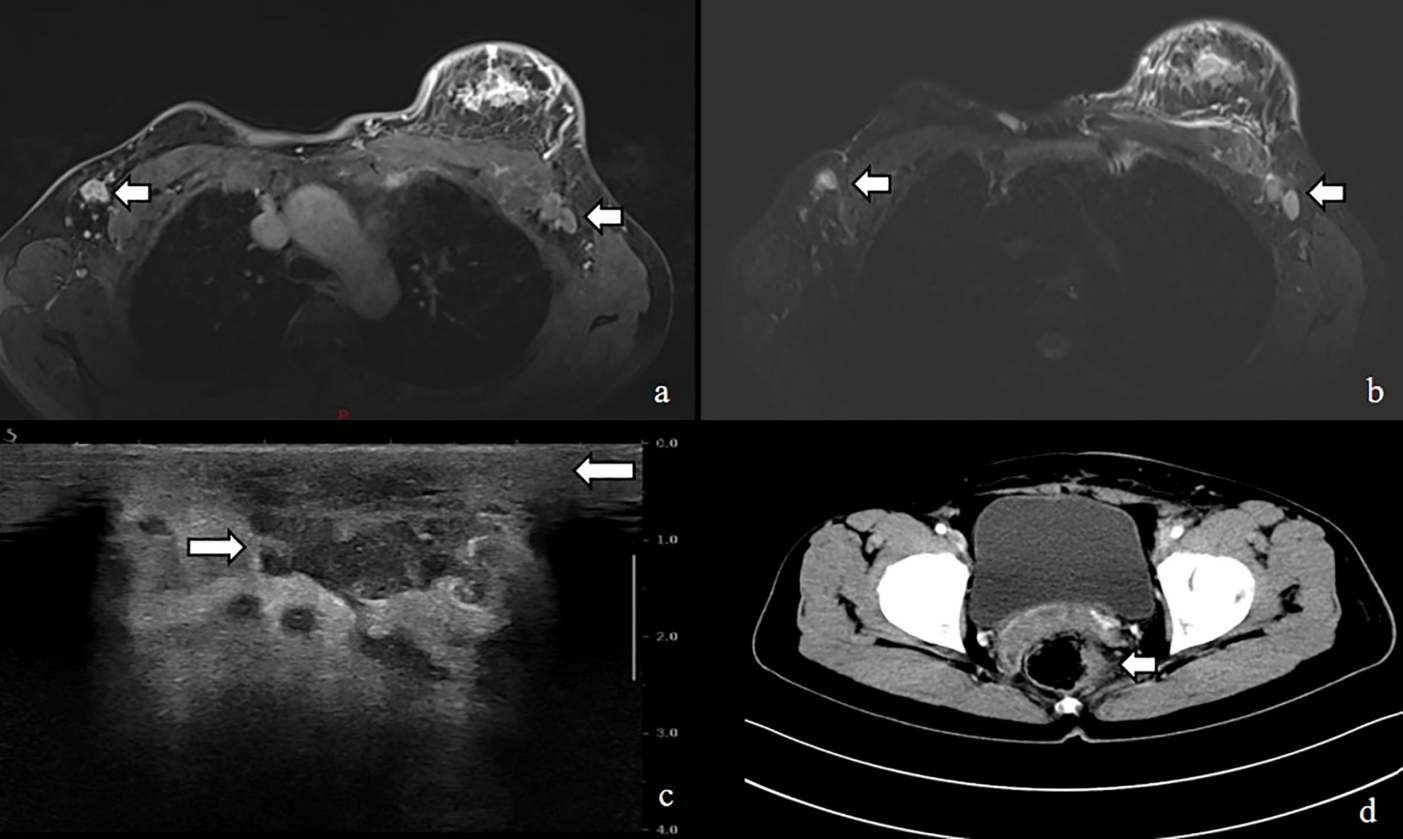
MRI image shows **(A, B)** enlarged left breast with multiple foci of scattered abnormal enhancement and enlarged lymph nodes in the left axilla, Ultrasound shows **(C)** thickened subcutaneous soft tissue in the left breast and multiple irregular hypoechoic areas, CT shows **(D)** uneven thickening and enhancement of the rectal anastomosis wall with small nodules in the adjacent peri-intestinal space.

A breast color doppler ultrasound revealed edema and thickening of the subcutaneous soft tissue in the patient’s left breast, along with multiple irregular low echo images in the thickened area. The largest image measures 21 x 10 mm in extent. The patient’s chest CT confirmed an enlargement in the volume of the left breast, irregular thickening of the skin, and the presence of soft tissue shadows with unclear boundaries. Furthermore, the multiple swollen lymph nodes previously detected in the breast color ultrasound were also confirmed by chest CT. Fortunately, the patient’s head CT, abdominal ultrasound, and gynecological ultrasound did not reveal any abnormal changes, but the patient strongly refused to undergo the mammogram examination due to her complaint of being unable to tolerate the pain associated with it (**Figure 3**).

To determine the nature of the patient’s breast lump, we performed a rough needle puncture. Routine pathological showed that the lump we took showed an adeno-tubular arrangement with large nuclei and heteromorphism, which was considered to be an invasive carcinoma, while immunohistochemistry suggested ER(-), PR(-), HER-2(0), GATA-3(-), CDX-2(+), CK20(+), Villin(+), Ki-67(+,70%) (**Figure 1**).

Based on the patient’s medical history and positive rectal cancer marker on immunohistochemistry, we suspected that the breast mass was of rectal origin. To investigate further, we performed an abdominal CT examination which revealed bowel wall thickening at the anastomotic orifice, nodules in the adjacent peri-intestinal space, and multiple enlarged lymph nodes in the left inguinal region, parietal iliac vessels, and retroperitoneum. Due to these findings, we strongly recommended that the patient undergo an enteroscopy and biopsy to confirm the diagnosis. However, the patient declined the procedure due to economic constraints and concerns about discomfort.

**Figure 4:**
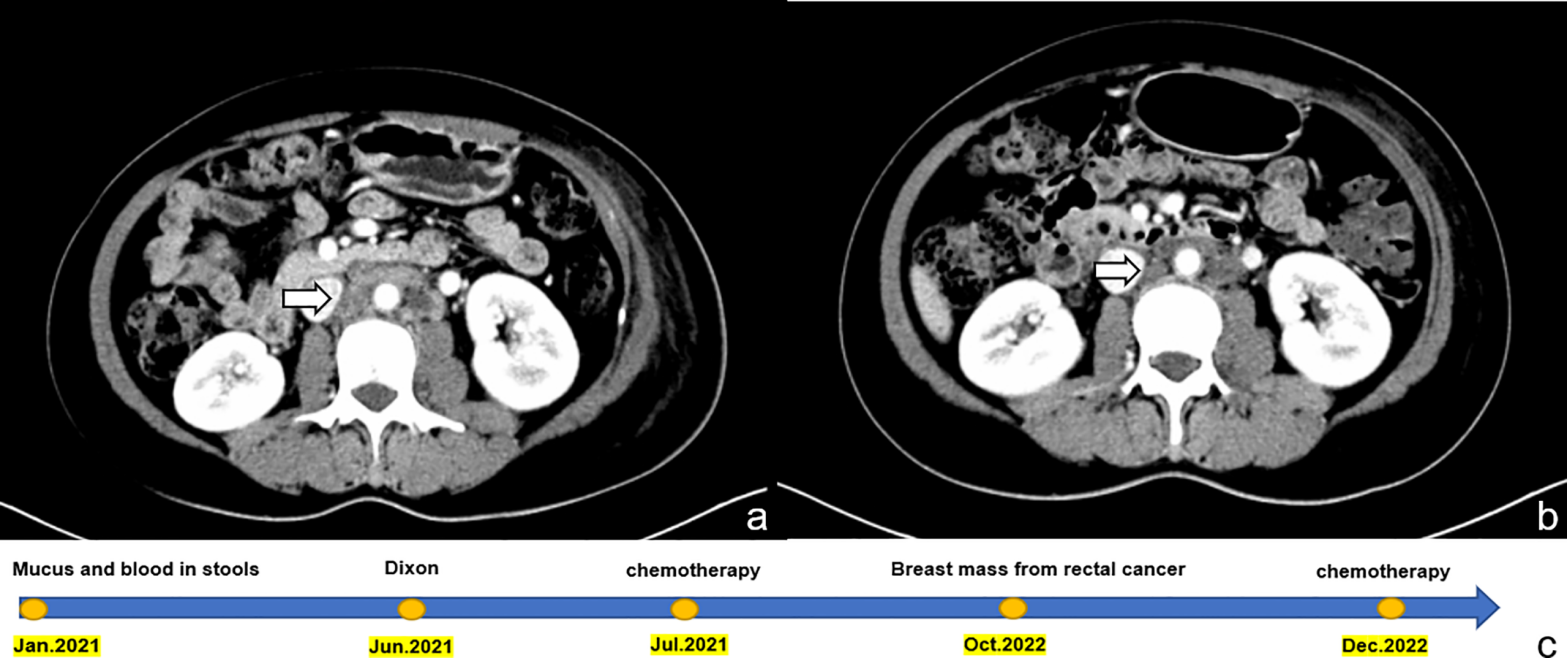
Shows a comparison of abdominal CT images before and after chemotherapy in the patient, and a timeline of the patient’s treatment process. **(A)** displays the image prior to chemotherapy, with enlarged lymph nodes indicated by the arrow. **(B)** displays the image after three courses of chemotherapy, with the same lymph nodes indicated by the arrow now visibly reduced in size. This visual representation highlights the effectiveness of chemotherapy in reducing the size of the lymph nodes. **(C)** timeline of the patient’s treatment process.

Although we did not obtain the results of the enteroscope, our multidisciplinary team (MDT) team, evaluated the patient’s medical history and physical examination, combined with the patient’s imaging examination and pathological findings, and eventually diagnosed the patient as having recurrent rectal cancer with a breast mass as the initial symptom. Given the suspected origin of the breast mass from the alimentary canal, the patient was transferred to the Department of Gastrointestinal Oncology for treatment. As our department specializes in breast surgery, we deemed it appropriate to transfer the patient to a department that could provide more specialized care for her condition. The patient was treated with a chemotherapy regimen of irinotecan 260 mg and fluorouracil 2.25 g combined with cetuximab 700 mg intravenous drip. The patient was last evaluated on February 21, 2023, the patient underwent an abdominal CT scan which showed that several small nodules with a short diameter of less than 1 cm in the adjacent peri-intestinal space were smaller than before, and the enlarged lymph nodes in the left inguinal region, adjacent to the iliac vessels and the retroperitoneum were slightly reduced (**Figure 4**). These findings suggest a positive response to the chemotherapy treatment.

## Discussion

Breast tumors of non-breast origin are rare, accounting for only a small proportion of all breast tumors (approximately 0.4%-5.1%). While metastases to the breast are known to occur in a variety of primary cancers, including lymphoma, lung cancer, and melanoma, rectal cancer as the origin of a breast mass is an extremely rare occurrence (1–4). Rectal cancer is the third most common cancer worldwide, with approximately 20% of patients presenting with distant metastases at the time of initial diagnosis, but such metastases are typically found in the lymph nodes, liver, or lungs (5, 6). Because of the differences in the follow-up and management of the two diseases, a definite diagnosis of these extremely rare cases is the key to the whole process.

Patients with breast metastases from rectal cancer present a diagnostic challenge for clinicians as there is no specific non-invasive method to confirm the diagnosis. Clinical manifestations are non-specific and usually include palpable breast lumps and axillary fossa lymphadenopathy. Skin changes such as redness and swelling may also be present, but these can also be seen in advanced breast cancer. Diagnosis based on clinical manifestations alone is difficult (7–9). Unfortunately, patients with breast metastases from rectal cancer do not show any specific imaging characteristics, and previous literature reports have shown that a significant number of patients are misdiagnosed with primary breast cancer or benign breast diseases based on imaging alone (10, 11).

When evaluating breast masses on ultrasound, it is important to distinguish those that originate from non-breast tissues from breast cancer. On ultrasound images, breast masses that originate from non-breast tissues tend to appear as well-defined, round, or oval, hypoechoic masses. They can be single or multiple and may occasionally appear minimally micro-lobulated. In contrast, breast cancer on ultrasound images is typically observed as a solid mass with irregular borders, microlobulations, or a spiculated appearance. Additionally, calcifications may appear as bright white spots in breast cancer cases. Breast masses that arise from non-breast tissues can appear as non-specific occupying lesions on mammography. These lesions may be solitary or multiple and usually lack calcifications. Additionally, diffuse opaque structural deformities may be observed in one or both breasts. In contrast, breast cancer typically appears as masses or clusters of microcalcifications on molybdenum target images. These small mineral deposits in breast tissue may be accompanied by irregular borders, microlobulations, or a spiculated appearance (10, 12–18).

Diagnosing breast metastases from rectal cancer is an uncommon and challenging task that typically requires routine pathology and immunohistochemistry. It is crucial to provide the pathologist with the patient’s complete medical history at the time of presentation. In a retrospective study of 85 non-breast-derived breast tumors, some cases of misdiagnosis occurred due to the pathologist’s inadequate knowledge of the patient’s past medical history. Therefore, emphasizing the importance of proper documentation and communication of past medical history is vital for accurate diagnosis and treatment (11).

Rectal cancer metastasizing to the breast is a rare occurrence, and there is limited understanding of its pathogenic mechanism. The presence of such metastasis indicates widespread dissemination and is associated with an unfavorable prognosis. Due to the scarcity of reported cases, it is challenging to determine the exact incidence of this metastatic pattern. To address this, we conducted a comprehensive analysis of existing literature, including 20 previously reported cases along with our own case.

Among the reported cases, a total of 20 patients had rectal cancer metastasizing to the breasts. The average age of these patients was 43.15 years, with the majority being females (**Table 1**). Only three male patients were reported. In 40% of the cases, metastasis was observed exclusively in the breast. In 45% of the cases, metastasis was observed in the left breast, consistent with our case, while in 15% of the cases, both breasts were affected. The onset of metastasis varied, with reports ranging from as early as 2 months to as late as 7 years. In our case, metastasis was diagnosed within 18 months from the initial diagnosis of rectal primary.

**Table 1:** Reported cases of rectal cancer metastasis to breast.

Study	Age	Sex	Metastasis	Time of detection of breast metastases	Location
Our study	41	Female	Breast	18months	Left
Alexander, H.R (19).	28	Female	Breast/Lung	11months	Right
Lal, R (20).	69	Female	Breast/Skin/Lung/Brain	1year	Left
Mihai, R (21).	53	Female	Breast/Skin	5years	Left
Hisham, R.B (22).	32	Female	Breast/Spine/Left eye/Orbit.	10months	Left
Wakeham, N (23).	45	Female	Breast/Liver/Lung	2 years	Bilateral
Li, H.C (24).	54	female	Breast/Lung/Skull base/Neck soft tissue	>2months	Right
Singh, T (1).	42	Female	Breast/Liver/Brain	11months	Right
Wang, T (7).	38	Male	Breast/Liver	7 years	Right
Sanchez, L.D (25).	36	Female	Breast	4months	Left
Makhdoomi, R (9).	28	Female	Breast	9months	Bilateral
Ahmad, A (26).	28	Female	Breast/Liver	0	Right
Aribas, B (27).	21	Female	Breast/Skin	10months	Bilateral
Shah, M (28).	49	Female	Breast	4months	Left
Hejazi, S.Y (29).	47	Female	Breast	3 years	Left
Hsieh, T.-C (30).	44	Female	Breast/Liver	7months	Right
Cheng, X (31).	57	Male	Breast	5months	Right
Wang, D.-D (32).	59	Female	Breast	16months	Left
Gur, E.O (33).	47	Male	Breast	2 years	Bilateral
Dai, Y (34).	45	Female	Breast/Lung	3 years	Left

In pathology, breast metastasis diagnosis relies on several histological features, such as well-defined margins, the absence of ductal carcinoma in situ, and no calcifications. However, even with the patient’s medical history, making a definitive diagnosis through conventional pathology can be challenging due to the similar growth patterns between metastatic carcinoma and breast cancer. Additionally, rare primary breast tumors, such as primary signet-ring cell carcinoma (SRCC), can be easily confused with metastatic signet-ring cell carcinoma, further complicating the diagnosis (2, 35). Distinguishing between rectal SRCC and other types of cancer based solely on pathological staining can be challenging. However, the good news is that colorectal SRCC can be distinguished using immunohistochemical markers such as negative Hep Par 1, homogeneous CDX2 nuclear positivity, and diffuse cytoplasmic positivity for MUC2 and MUC5AC in colorectal SRCC (36, 37).

Immunohistochemistry plays a critical role in diagnosing breast metastases from colorectal cancer by using specific markers to differentiate them from primary breast cancer. Two commonly used markers in gastrointestinal cancer diagnosis are cytokeratin proteins 20 (CK20) and cytokeratin proteins 7 (CK7). Typically, gastrointestinal cancer will show positive staining for CK20 and negative staining for CK7 (38). while primary breast tumors show the opposite staining pattern (39, 40). The literature suggests that CK20 expression in breast metastatic tumors is less than 6%, whereas the expression of CK7 in gastric metastasis of breast cancer can be as high as 83.34% (41). While CDX2 is useful in determining alimentary-derived tumors, it’s important to note that while most colorectal carcinomas are CDX2 positive, many gastric carcinomas are not. Furthermore, CDX2 can also be expressed in carcinomas originating from other sites, such as ovarian, endometrial, and lung cancers. Contrary to beliefs, studies have reported some expression of CDX2 in breast cancer, although at lower levels compared to gastrointestinal tumors. Nonetheless, CDX2 can still be a useful marker in distinguishing alimentary-derived tumors, including metastases from colorectal cancer, from primary breast cancer (40, 42, 43).

Although SATB2 expression is generally higher in breast, colon, and rectal cancer patients compared to their normal counterparts, it is utilized as a diagnostic marker for colorectal cancer in clinical settings (44–46). This is because SATB2 has been found to exhibit high sensitivity and specificity in colorectal adenocarcinoma, making it a valuable tool for diagnosing the disease. Studies have also suggested that a three-marker panel comprising SATB2, CK20, and CDX2 can improve the detection of metastatic colorectal cancer in liver biopsy tissues (47, 48).

As relatively specific markers for breast-derived tumors, GATA binding protein 3 (GATA3), mammaglobin, and gross cystic disease fluid protein 15 (GCDFP-15) are useful in determining the origin of the tumor (49). The expression level of GATA3 in breast cancer tissues is significantly higher than that of GCDFP-15 and mammaglobin, making GATA3 a particularly useful marker in identifying the origin of a tumor (50, 51). Moreover, GATA3 has higher sensitivity in identifying primary and metastatic breast cancers, and its expression rate in metastatic breast cancer is even as high as 96% (52). In addition, the combination of Villin and CDX2 markers can be used to infer the primary site of metastatic cancer. When both markers show positive staining, the tumor can be considered alimentary tract origin (53). The immunohistochemical results of this case were GATA-3 (-), CDX-2 (+), CK20 (+), and Villin (+). Based on these findings, the patient was eventually diagnosed with rectal cancer breast metastasis by our Multidisciplinary Team (MDT).

Systemic therapy is typically the preferred treatment for rectal cancer breast metastasis, while surgery is not usually recommended. However, metastasectomies are increasingly used for colorectal liver and lung metastases and have shown the potential to prolong survival in patients with well-controlled primary disease (24). Studies have also demonstrated that, when combined with effective systemic chemotherapy, metastasectomy can be an effective means of extending the survival of these patients (3, 25). Due to the rarity of this condition, there is no consensus on the best chemotherapy regimen to obtain definitive results. Considering the patient’s individual circumstances, a chemotherapy regimen consisting of 260 mg of irinotecan and 2.25 g of fluorouracil, in addition to a 700 mg intravenous drip of cetuximab, was administered. In addition, targeted therapy has emerged as a promising option for the treatment of metastatic colorectal cancer. Studies have shown that the use of targeted therapies can significantly improve the median overall survival in these patients, with a reported median survival of approximately 30 months (54).

The prognosis for patients with rectal cancer breast metastasis is poor, with a mean survival period of 14.9 months (30). Obviously, diagnosis is the most critical part of the entire process, which means that patients can receive early targeted treatment and improve their prognosis.

## Data availability statement

The original contributions presented in the study are included in the article/supplementary material. Further inquiries can be directed to the corresponding authors.

## Ethics statement

This case report has been approved by the Ethics Committee of Jiangxi Cancer Hospital. Written informed consent was obtained from the participant for the publication of this case report.

## Author contributions

JX, CY, and CL contributed to the writing of the manuscript text, reviewed the manuscript, and TY, FF, XZ, and CH contributed to the data collection process. WC, ZS, and MZ made a significant contribution to the manuscript by creating and formatting the figures, as well as reviewing the manuscript overall. All authors contributed to the article and approved the submitted version.
